# Conservation units and the origin of planted individuals of an endangered endemic species *Lobelia boninensis* in the Ogasawara Islands

**DOI:** 10.1038/s41598-024-78452-w

**Published:** 2024-11-09

**Authors:** Chikashi Hata, Chiharu Endo, Hiroshi Tanaka, Miho Hiruma, Maiko Kumamoto, Izumi Takenaka, Takashi Makino, Kento Niinaka, Yoshihisa Suyama, Shun K. Hirota, Michimasa Yamasaki, Yuji Isagi

**Affiliations:** 1 Graduate School of Agriculture, Kyoto University, Kyoto, Japan; 2 Tokyo Metropolitan Ogasawara Islands Branch Office, Tokyo, Japan; 3 Graduate School of Life Sciences, Tohoku University, Sendai, Japan; 4 Graduate School of Agricultural Science, Tohoku University, Sendai, Japan; 5 Botanical Gardens, Osaka Metropolitan University, Osaka, Japan

**Keywords:** Endangered species, Ogasawara Islands, Conservation units, Genetic population structure, MIG-seq, Biodiversity, Conservation biology, Ecological genetics

## Abstract

The Ogasawara Islands, one of the UNESCO World Natural Heritage Site located about 1000 km south of Japan’s main island, harbor numerous endemic species, many of which are as endangered. One of the endemic plant species, *Lobelia boninensis*, found in the Mukojima, Chichijima, and Hahajima archipelagoes, is endangered due to predation. As part of conservation efforts, translocation is now underway, especially on Chichijima. However, we lack essential information, such as the genetic population structure, to develop appropriate translocation strategies for both wild and planted individuals. Here, we aimed to identify the conservation units and the origin of planted individuals by estimating the genetic population structure and phylogenetic relationships across all habitats of this species. We identified two distinct genetic clusters, indicating genetic differentiation between the northern and southern populations. The genetic population components detected at an isolated site on Chichijima showed a mixture of these distinct clusters, probably due to hybridization. The transplanted individuals in Chichijima were found to have originated from a population in Hahajima. These results suggest the presence of two distinct conservation units. Furthermore, the current translocation strategy poses a risk of genetic contamination between these units, highlighting the need for revised conservation management practices.

## Introduction

The Ogasawara Islands, a UNESCO World Natural Heritage Site, are typical oceanic islands located approximately 1000 km south of the main island of Japan. They possess a unique ecosystem that is inhabited by numerous endemic and endangered species. These islands are of great importance due to their high biodiversity, which has prompted intensive conservation efforts to preserve these species. In order to conserve endangered species, it is essential to understand the conservation unit taking into account the spatial genetic structure and genetic diversity. In recent years, the advent of reduced representation genome sequencing, such as RAD-seq^1^ and MIG-seq^2^ has enabled the widespread adoption of genomic analysis for conservation purposes^3^.

**Fig. 1:**
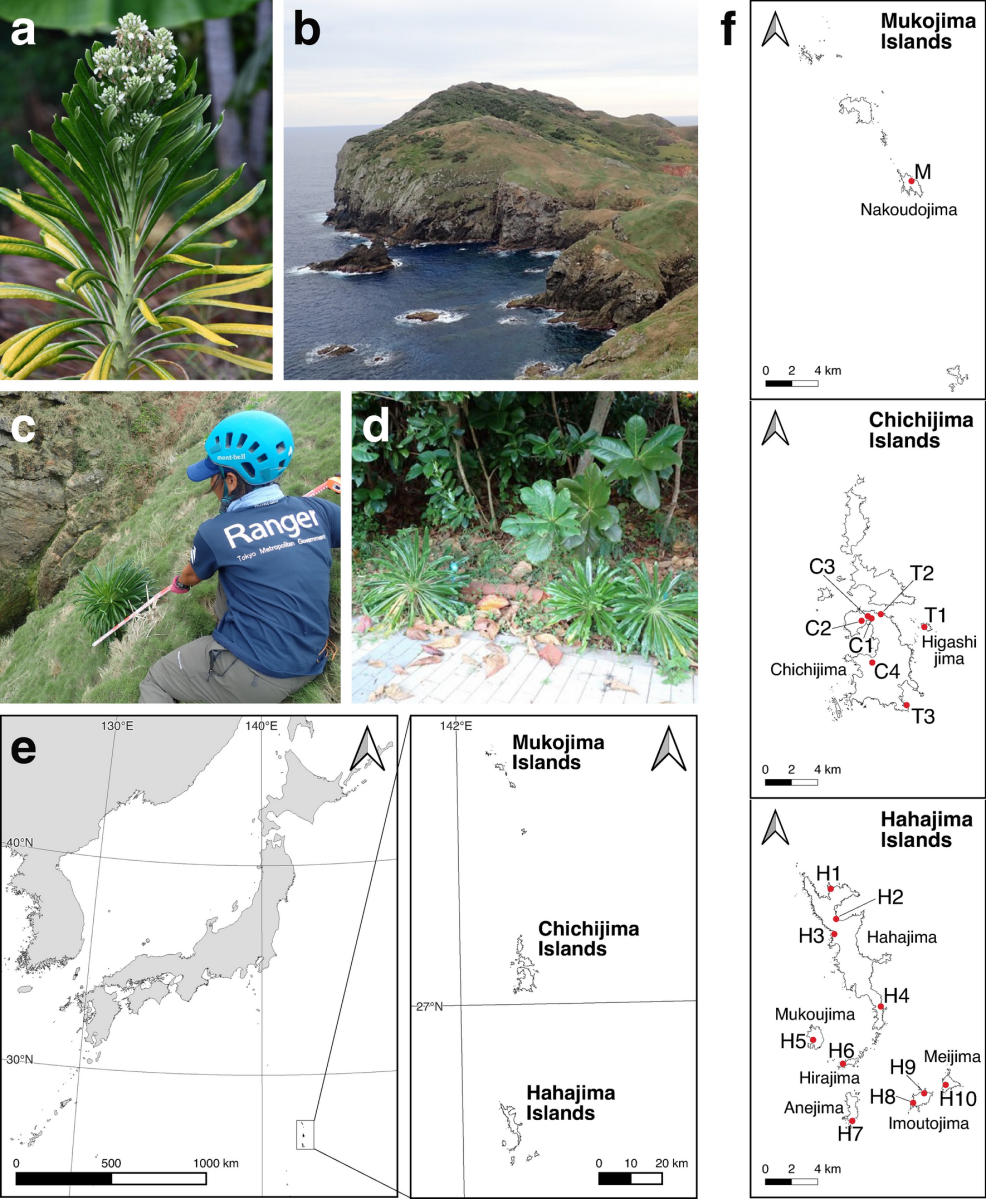
Habitats and sampling locations of *Lobelia boniensis*. (**a**) Flowering individual of *Lobelia boninensis*. (**b**), (**c**) Habitat of *L. boninensis* on Nakoudojima. (**d**) Planted individuals on Chichijima, (**e**) Geography of the Ogasawara Islands, situated approximately 1000 km south of the main island of Japan. (**f**) Sampling sites of each island.

With regard to the flora of the Ogasawara Islands, it has been documented that there are 125 endemic species, with 68% of these classified as endangered^4^. One prominent example of these endemic species is *Lobelia boninensis* (Campanulaceae), a notable species of the genus *Lobelia* that reaches a height of 2–3 m (see Fig. 1a), whereas the genus is predominantly composed of small herbaceous plants. It usually grows on cliffs along the coastline (Fig. 1b,c). The population sizes of this species have declined due to herbivory by feral goats and black rats^4^. Recently, it has been listed as Vulnerable in the Red List issued by the Ministry of the Environment and has been planted for conservation purposes on Chichijima (Fig. 1d). Most of its habitats are on unpopulated islands, including Nakoudojima in the Mukojima Islands, Chichijima and Higashijima in the Chichijima Islands, and Hahajima Island and its satellite islands (Fig. 1e,f). Particularly, on Higashijima Island, the number of wild individuals of this plant species increased after the eradication of the major herbivores, such as feral goats; however, they subsequently decreased due to feeding damage from black rats^5,6^. Recent documentation again indicates population recovery following the elimination of black rats in 2010^7,8^. Such historical events may influence population-specific conditions, such as genetic diversity. Although a previous study by Sugai et al.^9^ assessed the genetic diversity and population structure using four microsatellite loci for samples collected from several localities, a comprehensive understanding of the population genetic structure has been hindered. This is attributed to the limited information on individuals on Chichijima (wild and planted) and some of the affiliated islands of Hahajima. Furthermore, the actual population source of the planted individuals on Chichijima has remained unidentified.

The objective of this study was to investigate the population genetic structure, phylogenetic relationships, and genetic diversity across nearly all habitats of this species. To achieve this, we conducted reduced representation genome sequencing by MIG-seq (and RNA-seq for phylogenetic analysis) on a comprehensive set of samples, including both wild and planted individuals collected from nearly all habitats. Based on the results, we discuss the appropriate conservation units and the origins of the planted individuals.

## Results

### Genetic population structure

**Fig. 2:**
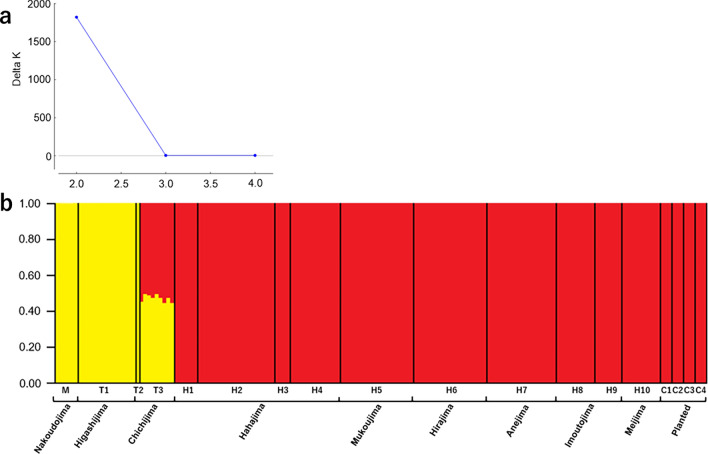
Results of Structure analysis for *Lobelia boninensis* based on 2394 SNPs. (**a**) DeltaK value calculated by STRUCTURE harvester. (**b**) Barplot on K = 2.

The analysis of genetic population structure based on SNPs (MIG-seq) data of *L. boniensis* in the Ogasawara Island revealed that the most probable number of inferred clusters (K) was K = 2 (yellow and red, see Fig. 2a). This demonstrates a clear genetic population structure, with the populations of Mukojima Islands (Nakoudojima, M) and Chichijima Islands (Higashijima and Chichijima, T1–T3) assigned a distinct cluster (yellow), separate from that of Hahajima Islands (H1–H10, red) (Fig. 2b). The individuals planted at four sites of Chichijima (C1–4) were found to belong to the cluster of Hahajima, rather than the cluster of Chichijima. A population in the south of Chichijima (T3) had mixed genetic clusters of Hahajima and Chichijima (Fig. 2b).

### Phylogenetic relationship

**Fig. 3:**
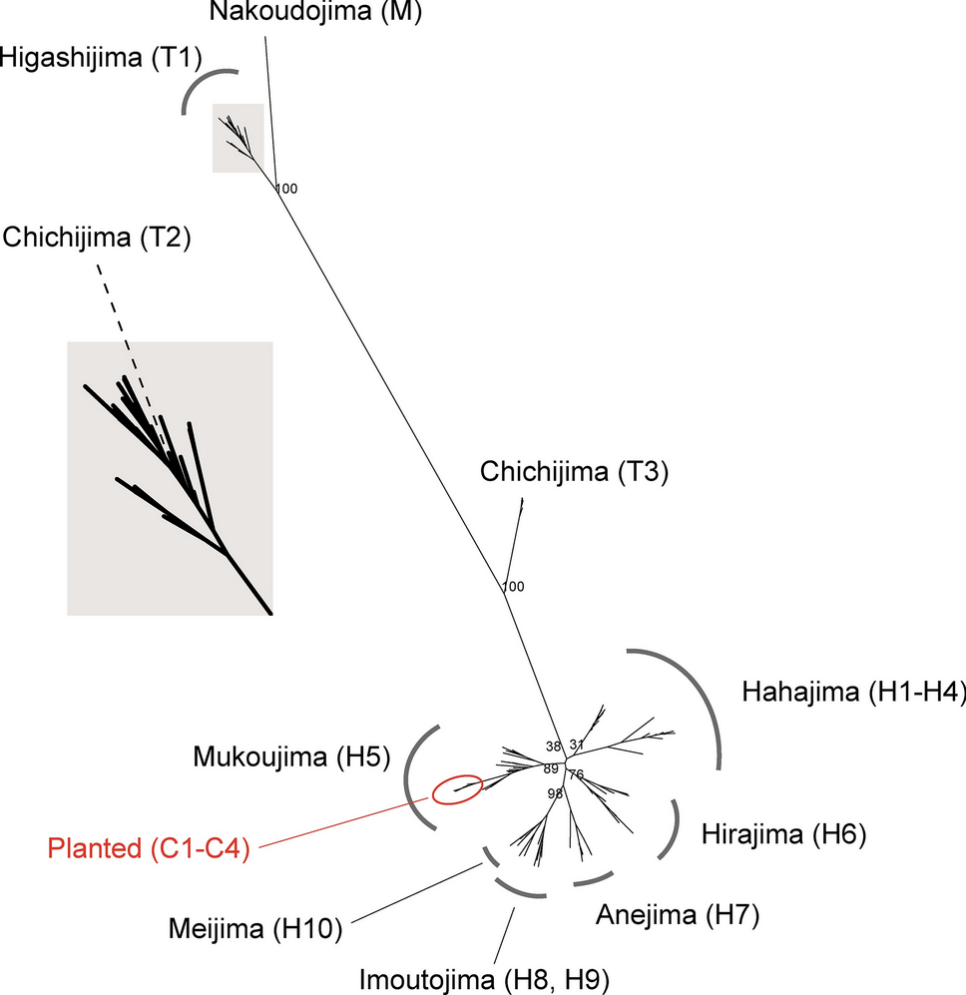
Phylogenetic relationship of *Lobelia boninensis* based on MIG-seq data. Bootstrap values are displayed at the main branching points, and the names of the islands are shown with their corresponding population codes. On the left, the top of the phylogenetic tree is enlarged to improve visibility.

**Fig. 4:**
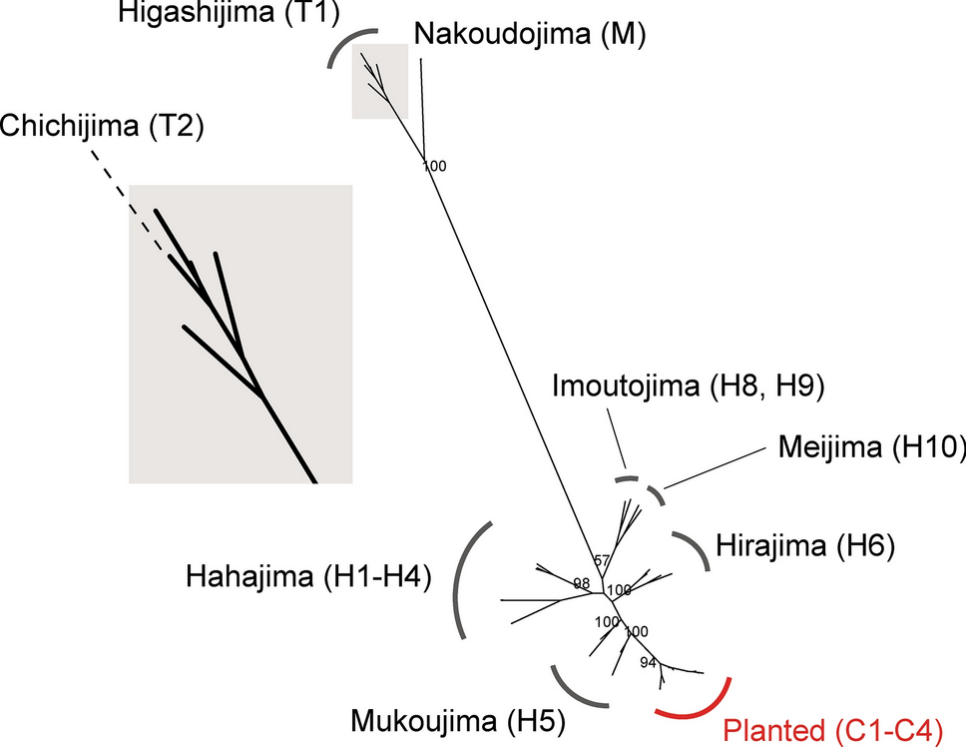
Phylogenetic relationship of *Lobelia boninensis* based on RNA-seq data. Bootstrap values are displayed at the main branching points, and the names of the islands are shown with their corresponding population codes. On the left, the top of the phylogenetic tree is enlarged to improve visibility.

The phylogenetic tree, constructed using MIG-seq data, comprised clades corresponding to geographic localities (Fig. 3). Individuals from Nakoudojima (M) of Mukojima Islands, Higashijima (T1) and Chichijima (T2) were separated into a distinct clade from a clade of the Hahajima Islands (H1–H10). Individuals from the southern part of Chichijima (T3) were situated between these two clades, and one from the northern part of Chichijima (T2) were included in the clade of Higashijima (T1). All planted individuals in Chichijima (C1–C4) were derived from a single lineage and were phylogenetically included in the clade of Mukoujima (H5). The phylogenetic tree constructed using RNA-seq data was found to be consistent with that based on MIG-seq data (Fig. 4). The entire set of samples analyzed was broadly divided into two clades: the clade of Higashijima (T1), the northern part of Chichijima (T2), and the island of Nakanoshima (M), and the clade of the Hahajima Islands (H1–H10) (Fig. 4). Furthermore, the planted individuals on Chichijima (C1–C4) again constituted a single clade with the samples from Mukoujima (H5) in the Hahajima Islands (Fig. 4).

### Genetic diversity at variant SNPs

**Fig. 5:**
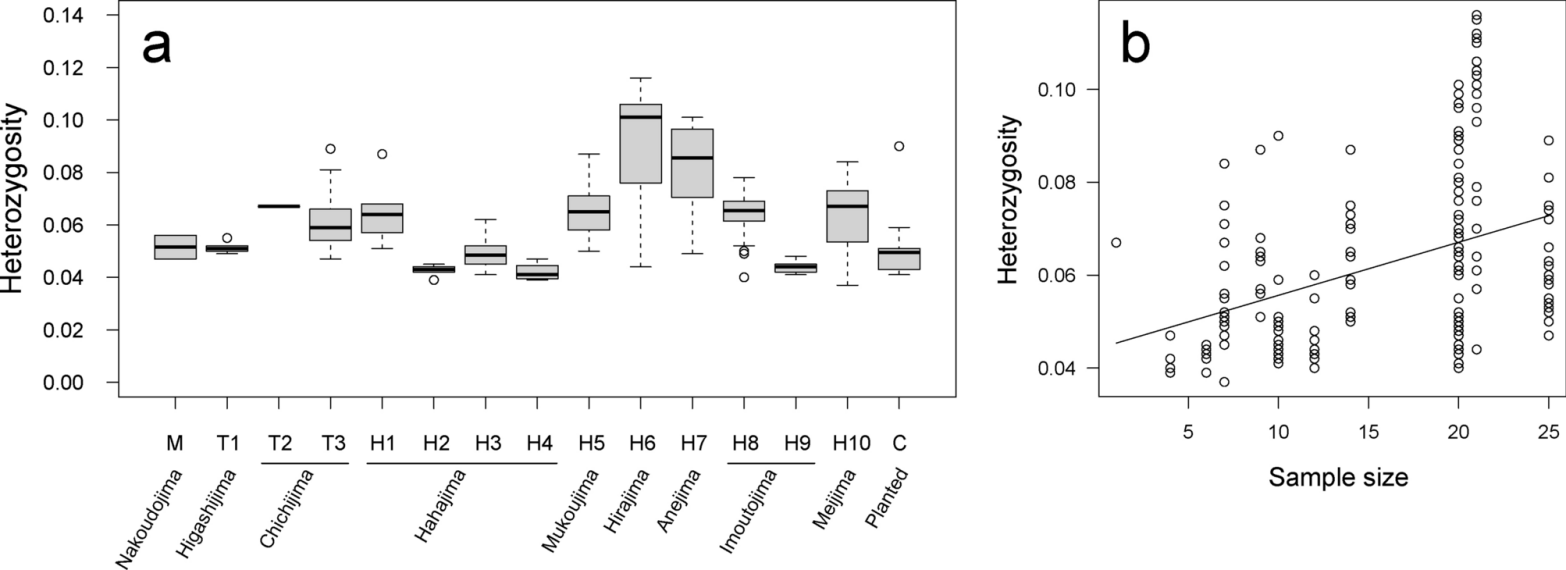
Comparisons of heterozygosity based on MIG-seq 7741 SNPs data. (**a**) Boxplot of heterozygosity values. The mean heterozygosity values significantly differ among local populations (Kruskal–Wallis, *P* < 0.001). (**b**) The relationship between sample size (the number of individuals per population used for the analysis) and heterozygosity values. There is a positive correlation between variables (GLM simple regression analysis, t = 5.63, *P* < 0.001).

By comparing the genetic diversity measured by heterozygosity within each sample (ranging from 0.037 to 0.116), we found significant differences in median heterozygosity values among local populations (Kruskal–Wallis, *P* < 0.001, Fig. 5a). However, the observed differences were affected by the analyzed sample sizes of the local populations; specifically, there was a positive effect of sample size on heterozygosity (GLM simple regression analysis, t = 5.63, *P* < 0.001; Fig. 5b).

## Discussion

The analysis of genetic population structure revealed a clear differentiation between the northern (Mukojima and Chichijima Islands) and southern (Hahajima Islands with its 5 satellite islands) parts of the Ogasawara Islands. The genetic population structure was consistent with the phylogenetic trees based on MIG-seq and RNA-seq data, as well as the results from a previous study^9^. These findings indicate that *L. boninensis* should be divided into two major conservation units: (1) the northern Ogasawara Islands (Mukojima and Chichijima islands; hereafter referred to as the “Chichijima Cluster”) and (2) the southern Ogasawara Islands (Hahajima Islands, including its satellite islands; hereafter referred to as the “Hahajima Cluster”).

The individuals planted on Chichijima (C1–C4) are suggested to have originated from a same clade as those on Mukoujima, one of the Hahajima Islands (Figs. 3, 4). Thus, the planted individuals on Chichijima can be classified as a distinct conservation unit, separated from the northern Ogasawara clade (i.e., the Chichijima Cluster), although it cannot be entirely ruled out that a natural population with similar genotypes may have once existed closer to this site. Here we highlight several concerns related to the planted individuals. First, the risk of genetic contamination through hybridization between the two conservation units. Although no wild individuals are currently growing in close proximity to the planted individuals, genetic exchange could still occur through natural dispersal mechanisms or other factors that facilitate gene flow. In addition to the dispersal of this species’ small seeds, pollen transfer may also be facilitated by pollinators, such as large bees (e.g., *Xylocopa ogasawarensis* and *Apis mellifera*). The pollinators are capable of long-distance dispersal over tens of kilometers^10^. We are particularly concerned the proximity of Higashijima (T1) to the planted site (4 km; see Fig. 1), and furthermore, the possibility of genetic exchange between Higashijima (T1) and northern Chichijima (T2) as suggested by our phylogenetic analysis (see Figs. 3, 4). Given the potential for long-distance seed and pollen dispersal, urgent management measures, such as replanting, may be necessary for the individuals planted in northern Chichijima.

The individuals at T3 (the south of Chichijima, Fig. 1) had mixed two genetic clusters, situated at an intermediate branch between the two clades of northern and southern Ogasawara Islands in the phylogenetic tree (see Fig. 3). These findings suggest the potential for hybrid individuals between the clusters. Nevertheless, further research is necessary to ascertain whether these individuals are indeed hybrids and to elucidate the underlying mechanisms.

This study did not detect an urgent decline in genetic diversity, as the values were at least around 0.04 (Fig. 5a). The persistence of genetic diversity may be attributed to: (1) reduced predation pressure (Tokyo Metropolitan Government, unpublished data) and (2) the germination of a soil seed bank that retains genetic diversity^11^. While the relationship between population size and genetic diversity is often predicted and evaluated under the traditional neutral theory, limitations of population genetic analysis based on neutral theory have been recently highlighted^12–14^, and Lewontin’s paradox^12^ suggests that genetic diversity does not increase as much as predicted by neutral theory. In the present case of *Lobelia boninensis*, the heterozygosity values significantly decreased with the sample size (Fig. 5b) possibly attributable to inbreeding occurring on a shorter time scale than assumed by neutral theory and Lewontin’s paradox. The observed positive correlation between sample size, representing the wild population size, and heterozygosity values, which are influenced by inbreeding, suggests the critical role of population size in preserving genetic diversity. One of the primary objectives of this study is to identify conservation units, and they are often recognized not only based on genetic differentiation at neutral sites but also by traits that are directly related to adaptation. Therefore, the SNPs used in this study are deemed effective in detecting conservation units within the taxon, irrespective of their neutrality. While the limitations of the neutral theory are acknowledged, the current data set provides a foundation for making informed conservation decisions.

In conclusion, our findings have three key implications for the conservation of *L. boninensis* in the Ogasawara Islands. First, the identification of two major genetic clusters suggests the necessity to establish two geographically delineated conservation management units for *L. boninensis*. Second, although the original population of individuals planted on Chichijima may have already disappeared, analysis of the existing populations provides compelling evidence that they originated from the Mukoujima, Hahajima Islands. To prevent genetic contamination and conserve the genetic lineage of Chichijima, it is crucial to replace the planted individuals with those from Chichijima Islands. Finally, although an urgent decline in genetic diversity was not found in this study, population size can be an important factor for maintaining genetic diversity. In particular, isolated populations with few individuals, such as on Nakoudojima, Mukojima Islands, will require continued conservation efforts. It is noteworthy that Nakoudojima exhibited greater genetic cohesion despite the larger distance between Nakoudojima and Chichijima compared to the distance between Chichijima and Hahajima (Figs. 1, 3). This highlights that geographic distance alone is not always a reliable predictor of genetic structuring. We lack information on the potential impacts of environmental factors, such as precipitation, on genetic processes and population characteristics. However, considering these factors may be valuable when developing conservation strategies for *L. boninensis*. Although we do not currently have evidence regarding whether hybrids possess lower fitness, the phylogenetic analysis suggests that the planted individuals (C) could pose a risk of artificial hybridization, potentially threatening the genetic integrity of the original lineages, such as those from northern Chichijima (T1 and T2). Furthermore, for T3 in the southern part of Chichijima, which exhibits genetic elements from both the Chichijima and Hahajima clusters, the mechanism underlying its genetic composition remains unclear. However, it is plausible that this composition arose through natural processes. The conservation strategy should prioritize minimizing genetic disruptions due to anthropogenic activities, including potential disturbances which may be caused by planted individuals in the northern part of Chichijima, and maintaining the distinct genetic characteristics of the defined conservation units.

## Materials and methods

### Sample collection

The collection and use of plant materials in this study were carried out in compliance with relevant institutional, national, and international guidelines and legislation. Necessary permissions and licenses were obtained from the relevant authorities (Ministry of the Environment and Forestry Agency, Japan). The plant specimens were identified by two of the current authors, Maiko Kumamoto and Yuji Isagi, and voucher specimens were deposited at Graduate School of Agriculture, Kyoto University. A total of 188 leaf specimens, including 176 natural individuals and 12 planted individuals, were collected from August 2021 to March 2022 (Table 1, Fig. 1). The sampling localities spanned the Mukojima Islands (Nakoudojima, M), Chichijima Islands (Higashijima, T1 and Chichijima, T2, T3), Hahajima Island (H1–H4), and the satellite islands within the Hahajima Archipelago (Mukoujima, H5; Hirajima, H6; Anejima, H7; Imoutojima, H8, H9; Meijima, H10; Table 1, Fig. 1). The planted individuals were collected from following locations: Okumura Sports Ground (C1), Ogamiyama Park (C2), Wildlife Research Institute (C3), and Subtropical Agriculture Center on Chichijima (C4) (Table 1, Fig. 1). This plant species typically grows on cliffs along the coastline (see Fig. 1b,c), and most of its habitats are on unpopulated islands that are difficult to assess. We collected as many samples as possible in collaboration with staff from the Natural Environment and Tokyo Park Rangers of the Civil Engineering Division, Ogasawara Branch office, Tokyo Metropolitan Government. The population size in the wild were estimated based on the observation survey by the staff (Table 1). For sites with fewer than 20 individuals, we achieved complete (100%) sampling (Table 1), while we aimed to collect approximately 20–30 individuals from sites with 20 or more individuals. Although this approach may introduce some uncertainty regarding exact coverage, this sampling set represents the characteristics of the entire population in a feasible manner. It enables analyses that can contribute to detecting conservation units, assigning individuals, and setting conservation priorities, all of which are essential for the biological conservation objectives of this study.

**Table 1:** Sampling sites, number of wild individuals and number of samples used for each analysis (MIG-seq, RNA-seq, STRUCTURE).

Site	Deposition number	Island	Longitude	Latitude	Collection date	Number of wild individuals	MIG-seq	RNA-seq	Structure
M	YIsagi1825	Nakoudojima	142.17866	27.63024	Nov. 26, 2021	7	7	4	6
T1	YIsagi 1712	Higashijima	142.24329	27.09326	Oct. 20, 2021	ca. 200	27	3	15
T2	YIsagi 1739	Chichijima	142.20988	27.10269	Oct. 20, 2021	1	1	1	1
T3	YIsagi 1879	Chichijima	142.22849	27.03934	Mar. 14, 2022	9	9	4	9
H1	YIsagi 1774	Hahajima	142.14331	26.70567	Nov. 7, 2021	6	6	3	6
H2	YIsagi 1852	Hahajima	142.14702	26.68477	Feb. 26, 2022	ca. 100	20	4	20
H3	YIsagi 1780	Hahajima	142.14542	26.67438	Nov. 7, 2021	4	4	3	4
H4	YIsagi 1740	Hahajima	142.18011	26.62367	Nov 11, 2021	14	14	3	13
H5	YIsagi 1804	Mukoujima	142.12766	26.60151	Nov 25, 2021	ca. 30	21	4	19
H6	YIsagi 1754	Hirajima	142.15023	26.58465	Nov 10, 2021	20	20	4	19
H7	YIsagi 1827	Anejima	142.15665	26.54495	Jan 17, 2022	ca. 50	20	4	18
H8	YIsagi 1784	Imoutojima	142.20363	26.55675	Nov 16, 2021	10	10	2	10
H9	YIsagi 1872	Imoutojima	142.21226	26.56317	Mar 15, 2022	7	7	2	7
H10	YIsagi 1794	Meijima	142.2289	26.56872	Nov 16, 2021	10	10	1	10
C1	YIsagi 1996	Chichijima	142.20267	27.099898	Aug 11, 2021	–	3	3	3
C2	YIsagi 1999	Chichijima	142.19494	27.098364	Aug 11, 2021	–	3	3	3
C3	YIsagi 2002	Chichijima	142.19982	27.101506	Aug 11, 2021	–	3	3	3
C4	YIsagi 2005	Chichijima	142.20258	27.069382	Aug 11, 2021	–	3	3	3

The samples were then dried with silica gel and used for DNA extraction. Samples for RNA extraction were fixed with RNA later (Qiagen, Hilden, Germany).

### MIG-seq analysis

DNA was extracted from each 1.5 cm^2^ dry leaf sample using the DNeasy Plant Mini Kit (Qiagen, Hilden, Germany) for MIG-seq library preparation. The MIG-seq analysis was conducted on extracted DNA samples following Suyama et al.^15^. The initial PCR amplification was conducted using primer set-1^2^ in 7 μl reaction volumes, containing 1 μl of template DNA, 2.24 μl of 1st PCR primers (a total of 16 primers, with 0.14 μl of each 10 μM primer), 3.5 μl of 2 × Multiplex PCR Buffer (Multiplex PCR Assay Kit Ver.2, Takara Bio, Kusatsu, Shiga, Japan), 0.035 μl of Multiplex PCR Enzyme Mix (Multiplex PCR Assay Kit Ver.2, Takara Bio) and 0.225 μl of deionized water, in an ABI Veriti 96-Well Thermal Cycler (Thermo Fisher Scientific, Applied Biosystem, CA, USA) with the following conditions: 94 °C for 1 min, followed by 30 cycles at 94 °C for 30 s, 38 °C for 1 min, 72 °C for 1 min, and a final extension at 72 °C for 10 min. 1st PCR products were purified/equalized, and short fragments (approximately < 250 bp) were removed using AMPure XP (Beckman Coulter, Brea, CA, USA) following the protocol of Hosomichi et al.^16^. In the 2nd PCR, index sequences and Illumina sequencing adaptors were added to the 1st PCR products. The fragments were amplified 6 μl reaction volumes, containing 1.6 μl of purified 1st PCR product, 1.2 μl of 5 × PrimeSTAR GXL Buffer (Takara Bio), 0.48 μl of each dNTP, 0.12 μl of PrimeSTAR GXL DNA Polymerase (Takara Bio) and 2.4 μl of 1 μM forward and reverse primer, with the following conditions:12 cycles at 98 °C for 10 s, 54 °C for 15 s, 68 °C for 1 min. The 2nd PCR products were mixed in equal volume. Purification and size selection (> 350 bp) were performed using AMPure XP (Beckman Coulter, Brea, CA, USA). Successful library preparation was confirmed using the Microchip Electrophoresis System for DNA/RNA Analysis MCE^®^-202 MultiNA with the DNA-2500 Reagent Kit (Shimadzu, Kyoto, Japan) according to the manufacturer’s protocol. Each library, at approximately 12 pM, was then sequenced on an Illumina MiSeq platform and MiSeq Reagent Kit v3 (150 cycles; Illumina, San Diego, CA, USA). We skipped the detection of the first 17 bases (SSR and anchor regions) in both Reads 1 and 2 using the “DarkCycle” option. The actual read lengths were defined as 80 bases for both reads.

Adapter sequences were removed from sequenced reads using trimmomatic v.0.38^17^. The cleaned data was input into Stacks v. 2.53^18^ to identify SNPs using parameter sets of M = 2, m = 3, and N = 4. Two individuals from Higashijima were excluded at this stage due to insufficient length for SNP calling. Following the procedure, 7741 loci of SNPs were obtained across 186 individuals, with a missing rate of 0.72616. In order to perform the population genetic analysis, the obtained SNPs data were filtered using TASSEL v.5.2.85^19^ to reduce the missing rate to less than 30% (0.29755) of the total SNPs data. This resulted in 23,94 loci, which left 169 individuals.

### Genetic population structure and phylogenetic analysis using MIG-seq data

The genetic variation obtained from reduced representation sequencing data, such as MIG-seq, includes both neutral (primarily) and non-neutral (relatively few) loci. In this study, we analyzed a sufficient number of loci from MIG-seq, which should reflect the neutral genetic variation, to apply for population genetic analysis, such as genetic diversity, Structure, and phylogenetic analysis. The genetic diversity of local populations was quantified as heterozygosity within each local sample. To examine the effects of sample size (i.e., the number of individuals included in the analysis) on the calculated heterozygosity, we performed a generalized linear model (GLM) simple regression analysis. Additionally, we compared the median heterozygosity among populations using a non-parametric analysis of variance (Kruskal–Wallis test). We conducted Structure analysis using the obtained MIG-seq SNPs data. A series of Structure runs in the STRUCTURE software v.2.3.4^20^ were conducted with a range of K values 1–5 with 10 replicates for each K value, with a burn-in period of 50,000 iterations and 100,000 MCMC iterations. The optimal K values were estimated using Structure Harvester^21^. We compared genetic diversity within each sample among local populations and along the sample sizes. The genetic diversity was quantified using GenAlEx (Genetic Analysis in Excel) software v.6.503^22^ to determine the frequency of heterozygous sites across variant sites of all samples.

A phylogenetic analysis was conducted using SNPs data with RAxML-NG v.1.1.0^23^. The GTR model was employed with a bootstrap value of 100. The resulting phylogenetic trees were subsequently visualized using FigTree v.1.4.4^24^.

### RNA-seq and phylogenetic analysis

A total of 43 individuals of *L. boninensis* were subjected to RNA extraction from fixed tissues in RNAlater (Thermo Fisher Scientific). RNA was extracted using the ISOSPIN Plant RNA Kit (Nippon Gene, Tokyo, Japan) in accordance with the manufacturer’s instructions. The RNA samples were then sequenced on Illumina HiSeq X sequencers at 150 nucleotide paired-end reads. De novo RNA-seq assembly was conducted using Trinity v2.10.0^25^ for paired-end reads derived from all individuals. The assembled transcripts were utilized as a reference sequence for *L. boninensis* following the removal of short contigs (< 150 bp).

To identify single nucleotide variations (SNVs) for each individual, paired-end reads were mapped by BWA v0.7.12^26^ to the reference of transcriptome reconstructed by Trinity. SNVs were identified using the SAMtools (Sequence Alignment/Map tools) software v.1.9^27^. It should be noted that only SNVs in loci with read depths greater than10 were included in the study. A total of 10,206 transcripts exhibited at least one SNV and were expressed in all individuals. The total base length that could be aligned across 43 individuals was 10,503,983 bp. The number of SNVs in the transcripts was 108,019. The transcript sequences were aligned using MAFFT v7.222^28^, and a maximum likelihood tree was constructed by RAxML v8.2.12^29^.

## Data Availability

Raw MIG-seq data are deposited at the DDBJ Sequencing Read Archive (DRA) with accession numbers DRA018233 (Submission).
